# Impact of Dose Reduction of Afatinib Used in Patients With Non–Small Cell Lung Cancer: A Systematic Review and Meta-Analysis

**DOI:** 10.3389/fphar.2021.781084

**Published:** 2021-11-29

**Authors:** Ziyu Wang, Xin Du, Ken Chen, Shanshan Li, Zhiheng Yu, Ziyang Wu, Li Yang, Dingding Chen, Wei Liu

**Affiliations:** ^1^ Department of Pharmacy, Peking University Third Hospital, Beijing, China; ^2^ School of Basic Medical Sciences and Clinical Pharmacy, China Pharmaceutical University, Nanjing, China; ^3^ Department of Pharmacy Administration and Clinical Pharmacy, School of Pharmaceutical Sciences, Peking University Health Science Center, Beijing, China; ^4^ College of Pharmacy, University of Nebraska Medical Center, Omaha, NE, United States; ^5^ Department of Obstetrics and Gynecology, Peking University Third Hospital, Beijing, China

**Keywords:** afatinib, non–small cell lung cancer, dose reduction, effectiveness, safety, meta-analysis

## Abstract

**Background and Aim:** As one of the second-generation epidermal growth factor receptor (EGFR)–tyrosine kinase inhibitors, afatinib brings survival benefits to patients with common and rare EGFR mutations. This study aimed to compare the effectiveness and safety of 30 and 40 mg of afatinib in patients with non–small cell lung cancer (NSCLC) using qualitative and quantitative analysis methods so as to provide reference for clinical medication.

**Methods:** The PubMed, Embase, ClinicalTrials.gov, Cochrane Library, China National Knowledge Infrastructure, and WanFang databases were thoroughly searched from inception to February 26, 2021. Two researchers independently screened the literature, extracted data, and evaluated the quality. RevMan and Stata 15.0 were used for meta-analysis.

**Results:** Twelve cohort studies including 1290 patients for final analysis were selected; of which, 1129 patients were analyzed to measure the effectiveness outcomes and 470 patients were analyzed for safety outcomes. In patients with non-brain metastasis, the progression-free survival of the first- or second-line treatment with reduced-dose afatinib was equivalent to the conventional dose. In terms of safety, the reduced dose could significantly lower the incidence of severe diarrhea and severe rash, but not the total incidence of diarrhea, rash, and all levels of paronychia.

**Conclusions:** The incidence of common serious adverse reactions was significantly lower with 30 mg of afatinib than with 40 mg of afatinib in patients with NSCLC. The effectiveness appeared to be similar to that in patients with non-brain metastasis. This study provides a reference for clinical dose reduction of afatinib.

**Systematic Review Registration:** [PROSPERO], identifier [CRD42021238043]

## Background

According to the International Agency for Research on Cancer’s (IARC) 2020 cancer report, lung cancer is currently the most common cause of cancer deaths (GLOBOCAN, 2020). Non–small cell lung cancer (NSCLC) accounts for about 85% of all lung cancers, and the 5-year survival rate for advanced patients was only 23% (Miller et al., 2019). In recent years, the drug treatment for NSCLC has developed from chemotherapeutic drugs to targeted drugs, wherein epidermal growth factor receptor-tyrosine kinase inhibitors (EGFR-TKIs) are targeted drugs that target EGFR for anticancer treatment. The EGFR-TKI therapy has shown significant survival benefits in patients with EGFR-mutant NSCLC. It has become the first-line treatment recommended by the guidelines of the National Comprehensive Cancer Network, the European Society for Medical Oncology, and the Chinese Society of Clinical Oncology (NCCN, 2021; ESMO, 2020; CSCO, 2020).

As a second-generation EGFR-TKI, afatinib can bind to the kinase domains of multiple subtypes in the EGFR family and inhibit the autophosphorylation of tyrosine kinases. Afatinib treatment has better progression-free survival (PFS) than the first-generation drugs and in patients with exon19 deletion mutation, which approximately accounts for 45% of EGFR mutations and showed better benefits (Yang et al., 2015; Park et al., 2016). However, the incidence of afatinib-related adverse reactions has been high and more serious because of the pan-target characteristics of afatinib. The most common adverse reactions are diarrhea, rash, and paronychia, which have a negative impact on the quality of life in patients. Afatinib can improve disease-related symptoms, such as chest pain and dyspnea, of patients with lung cancer compared with the placebo group. However, it increases diarrhea and loss of appetite at the same time, according to the patient-reported outcome analysis of clinical registration research LUX-Lung 1 (Hirsh et al., 2013).

According to the label, the recommended initial dose of afatinib is 40 mg, which can be reduced by 10 mg if not tolerated (Afatinib label, 2019). In clinical trials, the proportion of patients with dose reduction is 28–53% (Sequist et al., 2013; Wu et al., 2014), and more than half of the patients undergo dose modification due to adverse reactions in real-world studies (Kim et al., 2017; Cheema et al., 2019). It is an important clinical issue, but whether dose reduction can reduce the incidence of adverse reactions and achieve similar clinical effectiveness simultaneously remains elusive.

Some observational studies reported the effectiveness and safety of afatinib dose reduction, but no systematic review or meta-analysis integrated the existing study results. Therefore, this study aimed to compare the effectiveness and safety of 30 and 40 mg of afatinib in patients with NSCLC using qualitative and quantitative analyses to provide reference for clinical medication.

## Methods

This systematic review and meta-analysis was performed and reported according to the meta-analysis of observational studies in epidemiology (Stroup et al., 2000) and the preferred reporting items for systematic reviews and meta-analyses checklists (Liberati et al., 2009). The review protocol was registered in the International Prospective Register of Systematic Reviews and the registration number is CRD42021238043.

### Search Strategy and Eligibility Criteria

The search terms were related to “afatinib” and “non–small cell lung cancer.” The PubMed, Cochrane Library, EMBASE, ClinicalTrials.gov, China National Knowledge Infrastructure, and WanFang databases were searched to identify relevant publications in English and Chinese from inception to February 2021. The search syntax is provided in Supplementary Table SA.

The inclusion criteria were as follows: comparison of the effectiveness and safety of afatinib dose reduction in patients with NSCLC, age 18 years and above, using afatinib alone or in combination with other non–EGFR-TKI drugs. The exclusion criteria were as follows: doses were mixed when evaluating the effectiveness and safety of afatinib 30 and 40 mg, non-English or Chinese studies, no availability of full-text articles, letters, abstracts, meeting proceedings, and case reports. Two reviewers (WANG and DU) independently screened titles and abstracts for the eligibility of identified studies and then independently reviewed full-text articles. Disagreements were resolved by referring to a third reviewer (CHEN).

### Data Extraction

Two reviewers (WANG and DU) extracted data independently, using a predefined data extraction file. The following baseline characteristics were extracted from the included studies: first author, year of publication, study design, country in which the study was performed, study period, number of included patients, patient baseline characteristics and drug regimen of the intervention (30 mg of afatinib) and comparator (40 mg of afatinib) groups, and the effectiveness and safety outcomes of the intervention and comparator groups, such as the objective response rate (ORR), disease control rate (DCR), PFS, and incidence of common adverse events of all grades.

### Quality Assessment

Two reviewers independently assessed the methodological quality of included studies using modified Newcastle–Ottawa Scales (Ga Wells et al., 2021). Disagreements were resolved by consensus.

### Primary and Secondary Outcomes

The primary outcome of effectiveness was PFS. The secondary outcomes included the ORR and DCR. The ORR was defined as the sum of the proportions of complete response and partial response, and the DCR was defined as the sum of the proportions of complete response, partial response, and stable disease; evaluations were based on the Response Evaluation Criteria In Solid Tumors (RECIST) 1.1 (Eisenhauer et al., 2009). The primary outcome of safety is the incidence of adverse events of all grades (diarrhea, rash, and paronychia). The incidence and severity of all adverse events were documented according to the common terminology criteria for the same.

### Statistical Analysis

We used Stata 15.0 for statistical analysis of PFS and used Review Manager (Revman, version 5.3) for other statistical analyses. The dichotomous outcome was reported as the risk ratio (RR), the survival outcome was reported as the median survival ratio (MSR), and the data were analyzed using a random-effect meta-analysis based on the Mantel-Haenszel and DerSimonian—Laird model accompanying 95% confidence intervals (CIs). Statistical heterogeneity between studies was assessed using *I* ^2^ and *χ* ^2^ (test level *α* = 0.1) statistics. In the case of statistical heterogeneity, the sources of heterogeneity were either explored through the subgroup and sensitivity analyses or only descriptive analysis was performed. Publication bias was assessed using Egger’s test when at least 10 studies were included.

## Results

### Search

**FIGURE 1:**
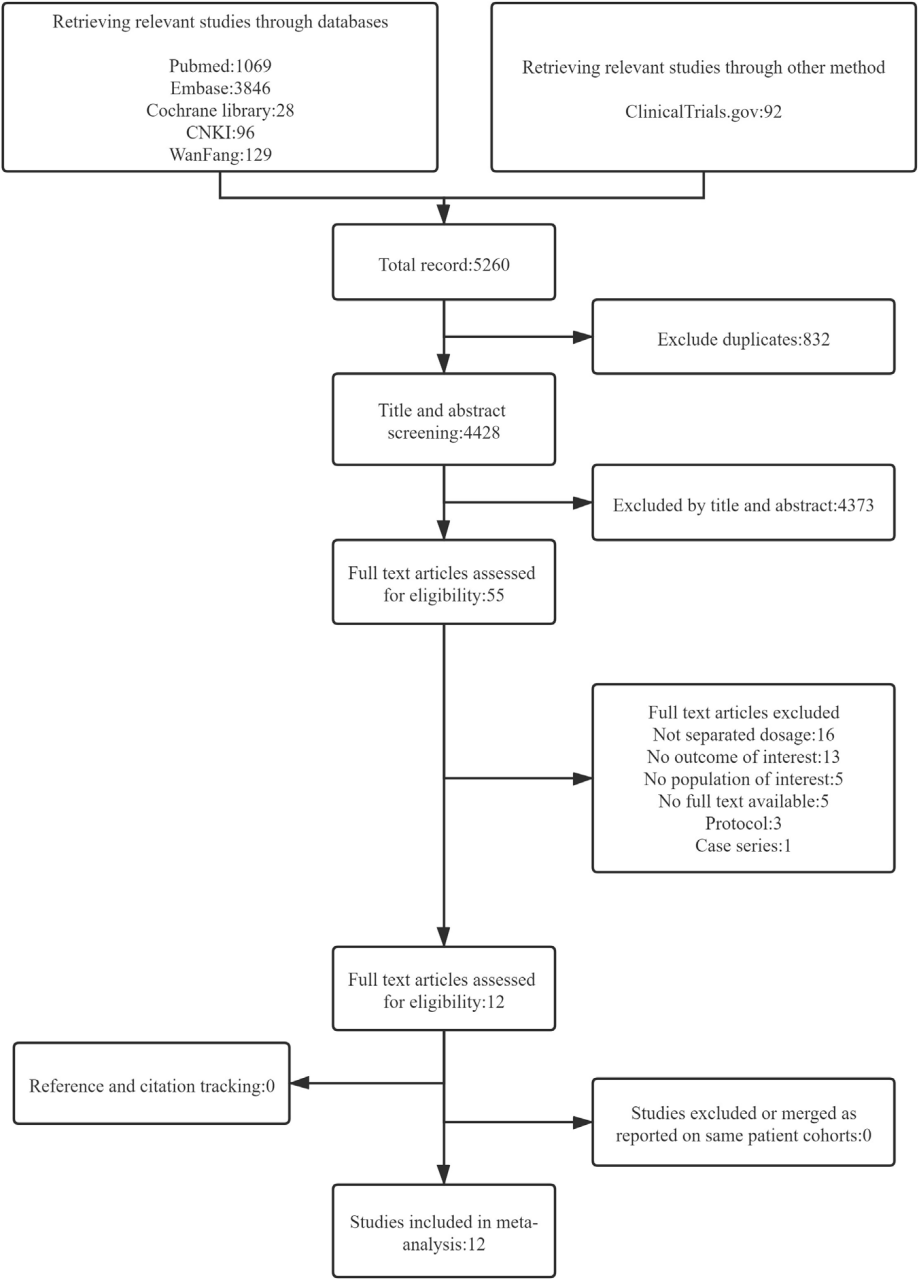
Flow diagram representing search and selection of studies comparing 30 versus 40 mg of afatinib treatment in patients with EGFR-mutant NSCLC.

The search strategy yielded 5260 citations. After removing duplicate citations, 4428 unique titles and abstracts were screened and 55 full-text articles were assessed for eligibility. Twelve cohort studies were eligible for inclusion, which comprised eight prospective studies and four retrospective studies (Arrieta et al., 2015; Moran et al., 2017; Yang et al., 2017; Lim et al., 2018; Ninomiya et al., 2018; Tan et al., 2018; Tanaka et al., 2018; Halmos et al., 2019; Tamura et al., 2019; Wang et al., 2019; Wei et al., 2019; Ko et al., 2021) (see Figure 1).

### Baseline Study Characteristics and Quality Assessment

Table 1 shows the baseline characteristics of included studies. In total, 12 studies, including 1290 patients, were selected for qualitative and quantitative analyses, with 362 patients in the 30-mg afatinib group and 928 in the 40-mg afatinib group. Moreover, 1129 patients were analyzed to measure effectiveness outcomes and 470 patients were analyzed to measure safety outcomes. The dosage regimen included afatinib administration only in nine studies and afatinib administration with concomitant medication in three studies. Most studies (9 studies, 75%) were launched in Asia, including 1 in Mexico, 1 in Spain, and 1 globally across 13 countries. The overall quality of the included studies was good. Among the 12 cohort studies included, 3 had a risk of bias in the assessment of prognostic factors and 2 did not match or were adjusted for a few plausible prognostic variables. Supplementary Table SB presents details of the risk-of-bias assessment for observational studies.

**TABLE 1:** Characteristics of the included studies.

Study and year	Region and study type	Overall no.^a^	Qualitative and quantitative analyses no.^b^		Age (year)		Sex (male %)		Adenocarcinoma (%)		ECOG ≤1%		Brain metastasis (%)		EGFR common mutation (exon19 del or exon21 L858R) %	
			30 mg	40 mg	30 mg	40 mg	30 mg	40 mg	30 mg	40 mg	30 mg	40 mg	30 mg	40 mg	30 mg	40 mg
afatinib only																
Arrieta, 2015 (Arrieta et al., 2015)	Mexico, prospective cohort study	84	39	26	Overall 59.3 ± 1.6^c^		Overall 70.2		NA		Overall 91.7		NA		NA	
Halmos, 2019 (Halmos et al., 2019)	Multicenter, prospective cohort study^e^	228	73	73	—	—	—	—	—	—	—	—	—	—	—	—
Lim, 2018 (Lim et al., 2018)	Taiwan, retrospective cohort study	158	44	114	65.8 (34.3–88.1) ^d^	61.2 (28.1–88.0) ^d^	28.1	41.1	100	100	89.1	94.7	32.8	31.1	68.7	86.8
Tamura, 2019 (Tamura et al., 2019)	Japan, prospective cohort study	1602	70	550	NA		34.5	43.7	NA		76.4	89.1	NA		NA	
Tan, 2018 (Tan et al., 2018)	Singapore, prospective cohort study	125	23	37	Overall 62 (26–86) ^d^		Overall 64		Overall 96.8		NA		Overall 33.6		Overall 91.2	
Tanaka, 2018 (Tanaka et al., 2018)	Japan, prospective cohort study	15	3	6	Overall 79 (75–87) ^d^		Overall 20		Overall 100		Overall 86.7		NA		Overall 93.4	
Wang, 2019 (Wang et al., 2019)	China, retrospective cohort study	60	19	41	58.1 (44.6–82.7) ^d^	57.2 (36.2–70.9) ^d^	47.4	51.2	100	100	100	100	31.6	43.9	68.4	70.7
Wei, 2019 (Wei et al., 2019)	Taiwan, retrospective cohort study	84	22	62	64.4 ± 12.1^c^	58.8 ± 9.7^c^	31.6	32.7	100	100	100	94.5	100	100	68.5	89.1
Yang, 2017 (Yang et al., 2017)	Taiwan, retrospective cohort study	48	29	19	67.3 ± 8.0^c^	60.6 ± 8.8^c^	21	63	100	100	76	84	28	21	100	100
afatinib + 15 mg/ kg bevacizumab																
Ko, 2021 (Ko et al., 2021)	Japan, prospective cohort study	16	14	2	Overall 63 (44–73) ^d^		Overall 31.25		Overall 100		Overall 100		NA		Overall 100	
Ninomiya, 2018 (Ninomiya et al., 2018)	Japan, prospective cohort study	19	14	5	67.5 (40–76) ^d^	65.0 (42–68) ^d^	50	60	100	100	100	100	50	40	100	100
afatinib +1 mg sirolimus																
Moran, 2017 (Moran et al., 2017)	Spain, prospective cohort study	39	12	4	Overall 58.9 ± 12.3^c^		Overall 38.5		Overall 84.6		Overall 97.4		NA		NA	

a Total number of people involved in the study, including dose groups other than 30 mg or 40 mg.

b Number of people included in the systematic review.

c Mean ± standard deviation.

d Median (range).

e Before and after self-control study.

NA, Not available.

### Effectiveness Outcome Measures

#### Progression-free Survival

PFS was reported in 6 studies involving 509 patients (Arrieta et al., 2015; Yang et al., 2017; Lim et al., 2018; Tan et al., 2018; Wang et al., 2019; Wei et al., 2019); median PFS data were presented as the MSR, and meta-analysis was performed in Stata 15 using the metan command. Significant heterogeneity was found between studies (*I* ^2^ = 85.4%). After applying various subgroup analyses, the heterogeneity was still high (*I* ^2^ = 70.1–89.1%). Therefore, only descriptive analysis was performed on the effectiveness outcome, as shown in Supplementary Figures S1-6.

Six studies used log-rank analysis to compare the median survival of patients using reduced and routine doses of afatinib, all with *p-value* > 0.05. Kim et al. (Kim et al., 2019) drew the Kaplan–Meier curve of 165 patients with the first-line use of afatinib and found that reducing the dose to 30 mg did not affect the PFS of the patients. However, Tan et al. (Tan et al., 2018) showed that in using afatinib as a first-line treatment in patients with stage IV NSCLC with brain metastases, 40 mg afatinib demonstrated better PFS than 30 mg afatinib (median PFS, 30 *vs.* 40 mg, 5.3 *vs.* 13.3 months, *p* = 0.04). Furthermore, the results were stable after being adjusted by the Cox regression model [hazard ratio (HR), 0.39 (0.15–0.99), *p* = 0.042]. However, Wei et al. (Wei et al., 2019) found no significant difference in PFS between the 30- and 40-mg afatinib groups in the same population (median PFS 30 *vs.* 40 mg, 9.1 *vs*. 12.9 months, *p* = 0.193). Table 2 shows the results of the six studies.

**TABLE 2:** Survival results of the six studies comparing patients using 30 and 40 mg afatinib.

Study	Population	Patient number		Study type	Median PFS (months)			HR (95% CI)	*p* value
		30-mg group	40-mg group		30-mg group	40-mg group	*p *value		
Arrieta, 2015 (Arrieta et al. 2015)	Stage Ⅳ second-line	39	26	Prospective	9.2 (4.5–13.8)	14.6 (7.2–22)	0.337	NR	NR
Lim, 2018 (Lim et al. 2018)	Stage IIIB + Ⅳ first-line	44	114	Retrospective	13.9 (NR)	16.8 (NR)	NR	NR	NR
Tan, 2018 (Tan et al. 2018)	Stage IIIB + Ⅳ first-line	23	37	Retrospective	10.7 (NR)	10.3 (NR)	0.367	0.63 (0.36,1.11)	0.113
	Stage Ⅳ with BM first-line	13	7		5.3 (3.1–10.8)	13.3 (6.6-UD)	0.040	0.39 (0.15–0.99)	0.042
Wei, 2019 (Wei et al. 2019)	Stage Ⅳ with BM first-line	15	30	Retrospective	9.1 (NR)	12.9 (NR)	0.193	NR	NR
	Stage Ⅳ with BM first-line with local treatment	4	25	Retrospective	7.7 (NR)	15.0 (NR)	0.193	NR	NR
Wang, 2019 (Wang et al. 2019)	Stage Ⅳ with BM	6	18	Retrospective	6.6 (4.5–8.8)	10 (0–22.6)	0.776	NR	NR
	Stage IIIB + Ⅳ first-line	10	29	Retrospective	5.2 (0.8–9.6)	14.5 (9.4–19.7)	0.101	NR	NR
	Stage IIIB + Ⅳ second-line	9	12	Retrospective	5.0 (2.5–7.5)	3.0 (1.3–4.8)	0.375	NR	NR
Yang, 2017 (Yang et al. 2017)	Stage Ⅳ first-line	29	19	Retrospective	15.6	14.8	0.842	0.40 (0.11–1.49)	0.172

BM, Brain metastasis; NR, not reported; UD, undefined.

#### ORR and DCR

**FIGURE 2:**
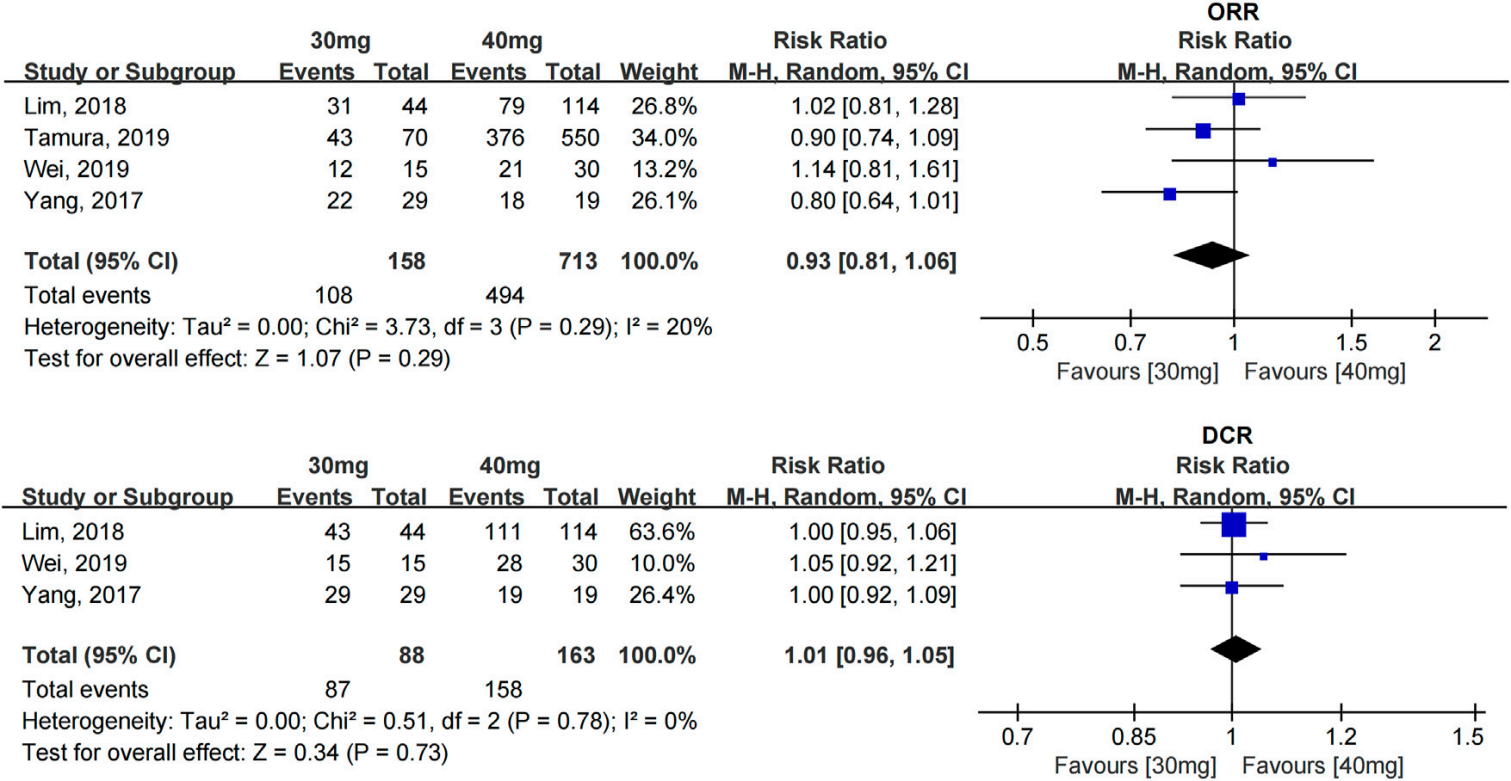
Forest plot of the ORR and DCR in the meta-analysis of patients treated with 30 and 40 mg afatinib.

The ORR and DCR were reported in four studies with 871 patients (Yang et al., 2017; Lim et al., 2018; Tamura et al., 2019; Wei et al., 2019), and all of them used afatinib as the first-line treatment. The heterogeneity between the studies was acceptable. No statistically significant difference in the ORR [RR = 0.93 (0.81, 1.06)] and DCR [RR = 1.01 (0.96, 1.05)] was found between the 40- and 30-mg afatinib groups (see Figure 2).

### Safety Outcome Measures

#### Diarrhea

The incidence of diarrhea was reported in seven studies with 454 patients (Yang et al., 2017; Lim et al., 2018; Ninomiya et al., 2018; Tanaka et al., 2018; Halmos et al., 2019; Wang et al., 2019; Ko et al., 2021), and no significant difference was found in the incidence of diarrhea between patients taking 30 and 40 mg afatinib [RR = 0.75 (0.52, 1.08), *p* = 0.35]. A subgroup analysis showed that when afatinib was used alone or combined with 15 mg/kg bevacizumab, no significant difference in the incidence of diarrhea was found among patients with a reduced dose compared with the conventional dose of afatinib. [RR = 0.75 (0.52, 1.08), *p* = 0.35], [RR = 0.91 (0.68, 1.21), *p* = 0.53].

**FIGURE 3:**
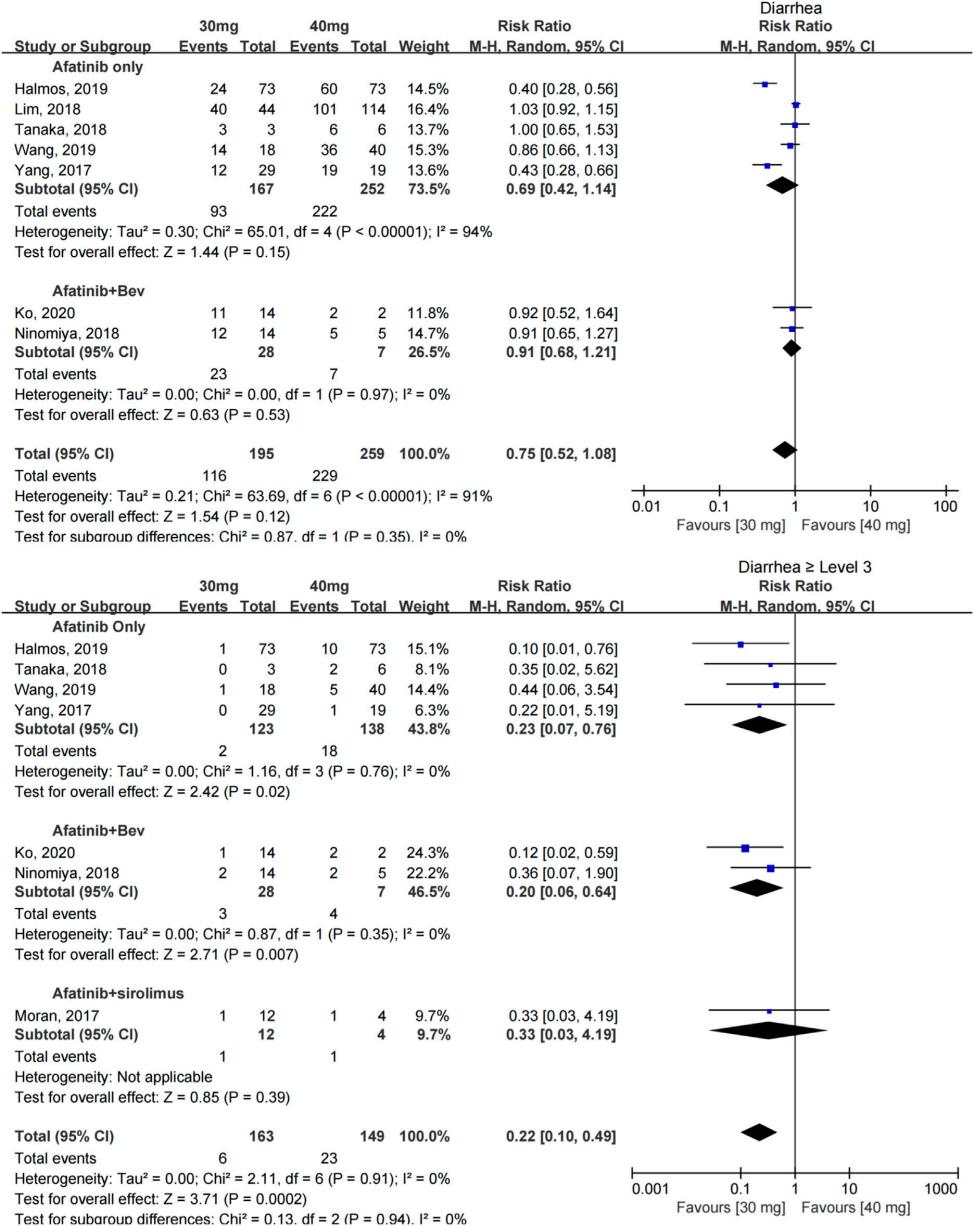
Forest plot of the incidence of diarrhea and ≥grade 3 diarrhea in the meta-analysis of patients treated with 30 and 40 mg of afatinib.

The incidence of severe diarrhea (≥grade 3) was reported in seven studies with 312 patients (Yang et al., 2017; Lim et al., 2018; Ninomiya et al., 2018; Tanaka et al., 2018; Halmos et al., 2019; Wang et al., 2019; Ko et al., 2021); no heterogeneity was found between these studies. Dose modification of afatinib to 30 mg significantly reduced the incidence of severe diarrhea [RR = 0.22 (0.10, 0.49), *p* = 0.0002] (see Figure 3).

#### Rash

The incidence of rash was reported in seven studies with 454 patients (Yang et al., 2017; Lim et al., 2018; Ninomiya et al., 2018; Tanaka et al., 2018; Halmos et al., 2019; Wang et al., 2019; Ko et al., 2021); 30 mg of afatinib did not show a reduction in the incidence of rash in patients [RR = 0.84 (0.67, 1.06), *p* = 0.15], and the subgroup analysis showed no significant difference whether afatinib was used alone [RR = 0.80 (0.60, 1.06), *p* = 0.12] or in combination with other drugs [RR = 1.03 (0.71, 1.49), *p* = 0.87].

**FIGURE 4:**
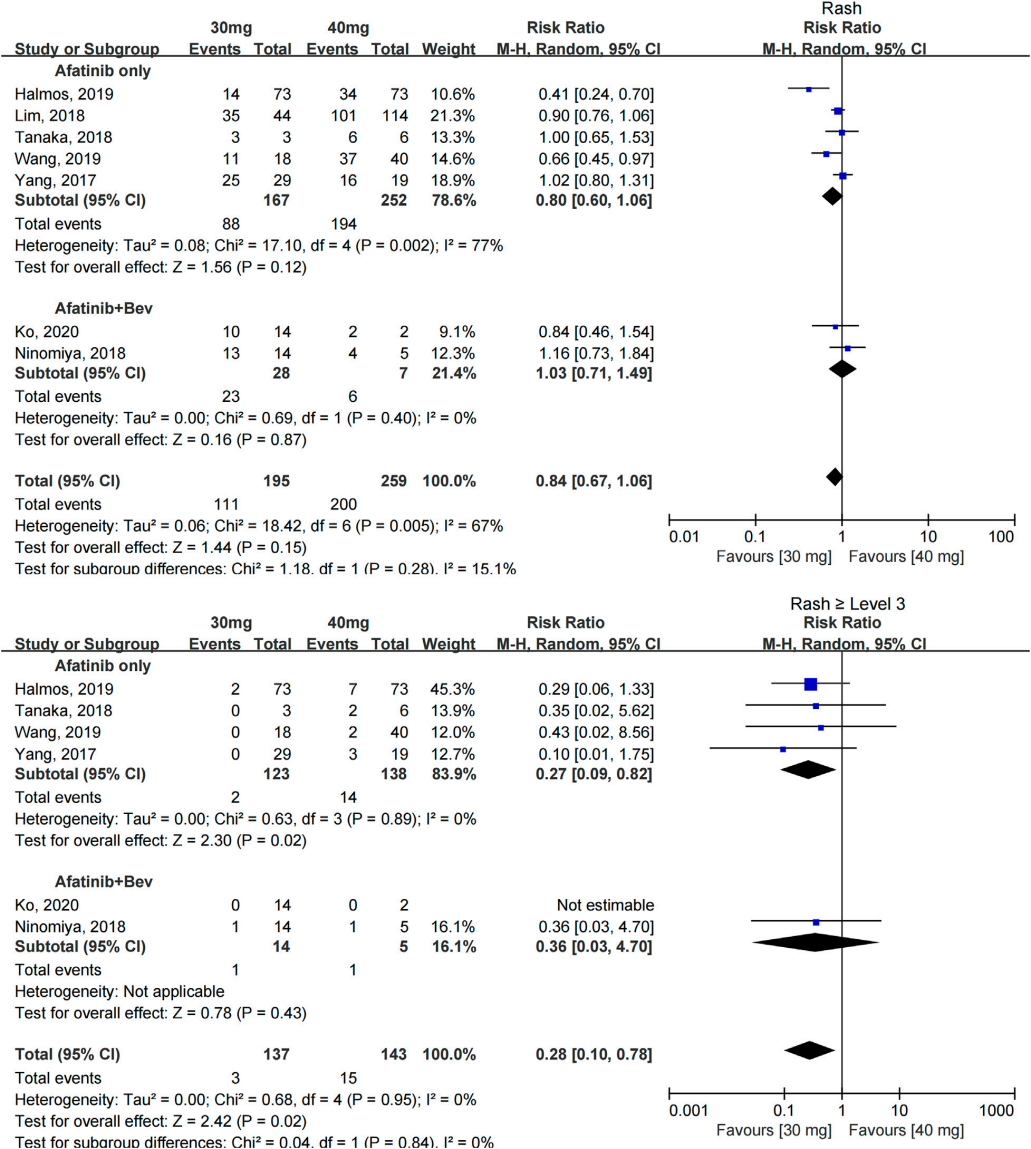
Forest plot of the incidence of rash and ≥grade 3 rash in the meta-analysis of patients treated with 30 and 40 mg of afatinib.

The incidence of severe rash (≥grade 3) was reported in 6 studies with 280 patients (Yang et al., 2017; Ninomiya et al., 2018; Tanaka et al., 2018; Halmos et al., 2019; Wang et al., 2019; Ko et al., 2021), and little heterogeneity was observed. Patients taking 30 mg afatinib had a lower risk of developing severe rash [RR = 0.28 (0.10, 0.78), *p* = 0.02] (see Figure 4).

#### Paronychia

The incidence of paronychia was reported in 6 studies with 308 patients (Yang et al., 2017; Lim et al., 2018; Ninomiya et al., 2018; Tanaka et al., 2018; Wang et al., 2019; Ko et al., 2021), and the incidence of paronychia in the two groups was similar [RR = 0.89 (0.67, 1.20), *p* = 0.45].

**FIGURE 5:**
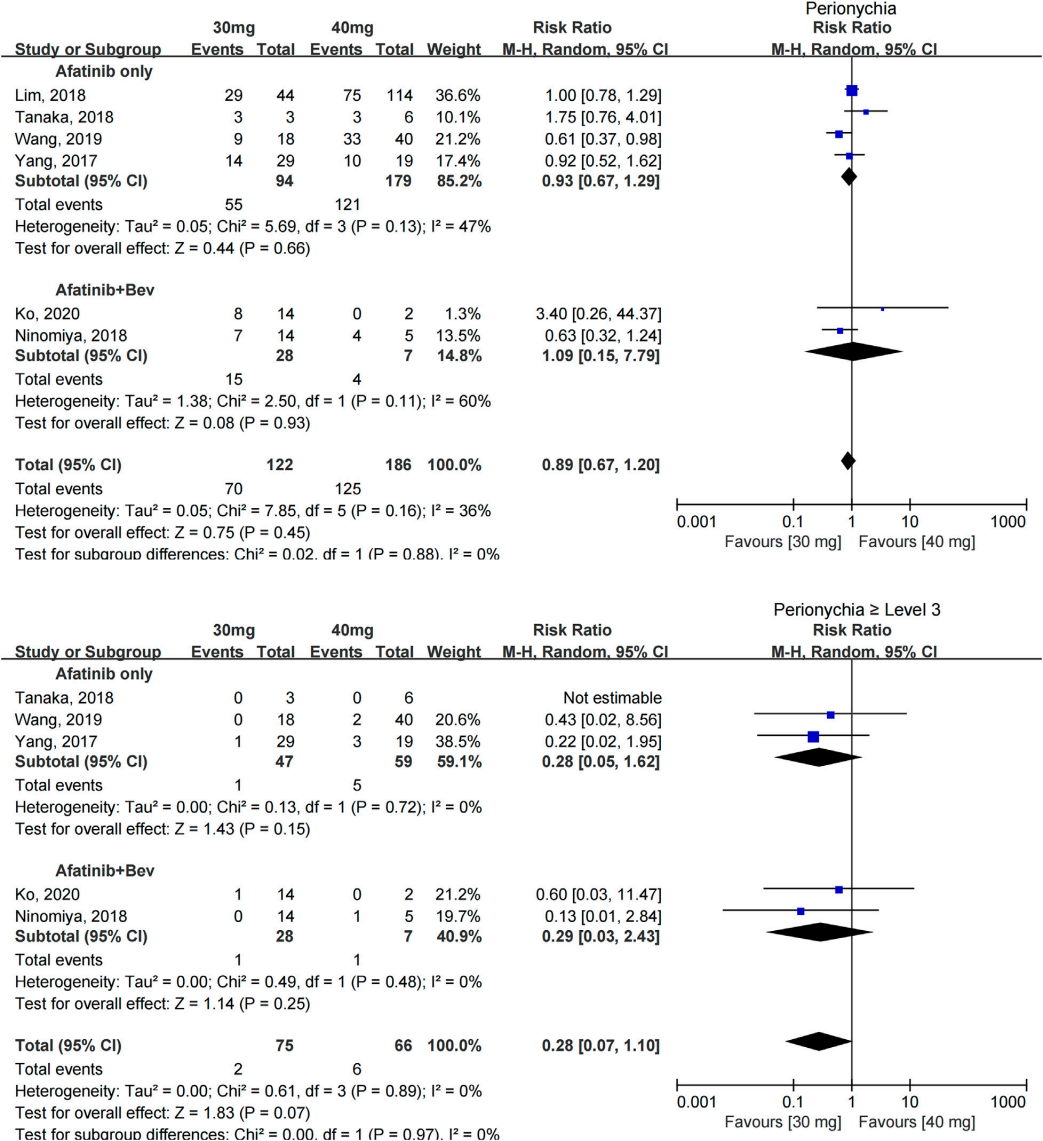
Forest plot of the incidence of paronychia and ≥grade 3 paronychia in the meta-analysis of patients treated with 30 and 40 mg afatinib.

The incidence of severe paronychia (≥grade 3) was reported in five studies with 141 patients (Yang et al., 2017; Ninomiya et al., 2018; Tanaka et al., 2018; Wang et al., 2019; Ko et al., 2021), and no statistical difference was found in the incidence of severe paronychia [RR = 0.28 (0.07, 1.10), *p* = 0.07] (see Figure 5).

### Sensitivity Analysis

For different outcome measures, sensitivity analysis was performed by excluding individual studies one by one. The results showed that meta-analysis results did not change in direction after excluding any study, indicating that these meta-analysis results were relatively stable.

## Discussion

The present systematic review and meta-analysis was the first study to compare the effectiveness and safety of patients with NSCLC using 30 and 40 mg afatinib. The results showed that in patients with advanced NSCLC without brain metastases, the PFS appeared to be equivalent between the 30- and 40-mg afatinib groups, irrespective of whether afatinib was used as a first-line or second-line treatment; however, the data were limited. No statistical difference in the ORR and DCR was found between the two groups of patients who used afatinib as the first-line treatment. In terms of safety, whether afatinib was used alone or in combination with other drugs, reduced-dose afatinib could significantly reduce the incidence of severe diarrhea and rash; however, the results did not indicate that dose reduction could reduce the incidence of paronychia at all levels.

Afatinib was approved by the U.S. Food and Drug Administration in 2013. As the second-generation EGFR-TKI, afatinib not only showed a better survival benefit for patients with common EGFR mutations (exon 19del and exon 21 L858R) but was also effective for patients with rare mutations (Banno et al., 2018; Chang et al., 2021). However, the incidence of adverse reactions was also significantly higher than that of the first- and third-generation drugs. The risk of diarrhea was the highest among the first- and third-generation drugs (RR = 38.88, *p* < 0.001) (Yin et al., 2021). Afatinib-related adverse reactions not only reduced the quality of life in patients but also increased their financial burden due to the cost of management (Villa et al., 2016; Subramanian et al., 2019). The median occurrence time of adverse reactions in patients using afatinib was mainly within 1 month from the initial medication (Cheema et al., 2017). Therefore, clinically, some doctors consider reducing the dose empirically at the first administration, while no definite evidence exists for clinical dose reduction currently. Of the 12 studies, 6 reported initial dose reduction and others included dose escalation trials (5 clinical phase I or II) and self-control analyses before and after the trial (1 study). After analyzing the patient characteristics of the included studies, the population characteristics of the 30-mg afatinib group were as follows. Gender: Six studies presented the baseline characteristics of patients with different doses, which revealed that more female patients took reduced doses than male patients. Especially in the study by Yang (Yang et al., 2017), the proportion of women taking 30 mg afatinib was twice that of those taking 40 mg afatinib (79 *vs*. 37%) (Miller et al., 2019). Weight: Only two studies reported the baseline information on body surface area (BSA) and weight; hence, the data are not summarized in Table 1. Patients who received reduced doses had significantly lower BSA (1.5 ± 0.2 *vs*. 1.7 ± 0.1, *p* = 0.0055) and smaller body weight (weight ≥ 60 kg, 19.1 *vs*. 33.4%). Age: Five studies presented the age of patients in different dose groups, revealing that patients in the 30-mg afatinib group were older. In the study of Lim et al. (Lim et al., 2018) and Yang et al. (Yang et al., 2017), the age of the 30-mg afatinib group was statistically significantly higher than that of the 40-mg afatinib group [65.8 (34.3–88.1) *vs*. 61.2 (28.1–88.0), *p* < 0.05; 67.3 ± 8.0 *vs*. 60.6 ± 8.8, *p* < 0.05]. The aforementioned population characteristics of reduced doses were basically consistent with the results of afatinib population pharmacokinetic studies (Freiwald et al., 2014), which indicated that women, less weight, low creatinine clearance, and high total protein levels tended to associate with greater drug exposure, and the bioavailability of afatinib in patients with a poor physical status increased.

Therefore, the theoretical basis for reduction in afatinib could be explained by the dose-exposure–response relationship. The steady-state plasma trough concentration after administering 30 mg afatinib was significantly lower than that of patients taking 40 mg afatinib daily (*p* = 0.02) (Nakao et al., 2019). The steady-state trough blood concentration of patients with serious adverse reactions requiring dose reduction or withdrawal could be twice that of other patients (Chiba, 2016). The blood concentration positively correlated with the severity of diarrhea in the early phase (*r* = 0.498, *p* < 0.05) (Hayashi et al., 2019). However, no afatinib exposure–response study was available to explore the relationship between blood concentration and effectiveness. In the phase I trial of afatinib, a high-dose intermittent administration of 55 mg afatinib daily (3 weeks for medication/1 week for rest) achieved a *C*_max_ about four times that of 40 mg afatinib daily, but no definite ORR exists (Marshall et al., 2013).

This study had several limitations. First, the number of relevant original studies was limited, further subgroup analysis based on population characteristics could not be performed, and the number of studies for each outcome was not enough to assess publication bias. Second, few included studies adjusted the outcome results for multivariate analysis, which might affect the accuracy of the final analysis results. Third, for the analysis of PFS, quantitative analysis could not be performed due to the obvious heterogeneity among the relevant studies.

## Conclusion

This study was the first systematic review and meta-analysis evaluating the effectiveness and safety outcomes of dose reduction of afatinib used in patients with NSCLC. The results showed that reduced-dose afatinib could significantly reduce the incidence of common serious adverse events. The effectiveness appeared to be comparable between the regular- and reduced-dose group, although the evidence was inadequate and of low quality. Further studies are needed to identify the appropriate population for initial dose reduction to provide more individualized and precise treatment to the patients.

## Data Availability

The original contributions presented in the study are included in the article/Supplementary Material; further inquiries can be directed to the corresponding authors.
