# Supplementary material for: Impact of Dose Reduction of Afatinib Used in Patients With Non–Small Cell Lung Cancer: A Systematic Review and Meta-Analysis
Source: Front Pharmacol. 2021 Nov 29;12:781084. doi: 10.3389/fphar.2021.781084 (PMC8666963; doi:10.3389/fphar.2021.781084)
Supplement: Supplementary file 1 [file DataSheet1.docx]

**Appendix A: Supplementary Figures and Tables**

Supplementary Table A Search syntax performed last on Feb 26th, 2021

| Database | Syntax |
| --- | --- |
| *PubMed（n=1069）* | ("non small cell"[All Fields] AND "lung"[All Fields]) OR "non-small-cell lung carcinoma"[All Fields] OR ("non"[All Fields] AND "small"[All Fields] AND "cell"[All Fields] AND "lung"[All Fields] AND "cancer"[All Fields]) OR "non small cell lung cancer"[All Fields] OR ("carcinoma, non small cell lung"[MeSH Terms] OR ("carcinoma"[All Fields] AND "non small cell"[All Fields] AND "lung"[All Fields]) OR "non-small-cell lung carcinoma"[All Fields] OR ("non"[All Fields] AND "small"[All Fields] AND "cell"[All Fields] AND "lung"[All Fields] AND "carcinoma"[All Fields]) OR "non small cell lung carcinoma"[All Fields]) OR ("carcinoma, non small cell lung"[MeSH Terms] OR ("carcinoma"[All Fields] AND "non small cell"[All Fields] AND "lung"[All Fields]) OR "non-small-cell lung carcinoma"[All Fields] OR "nsclc"[All Fields] OR "nsclc s"[All Fields] OR "nsclcs"[All Fields]) OR ("adenocarcinoma of lung"[MeSH Terms] OR ("adenocarcinoma"[All Fields] AND "lung"[All Fields]) OR "adenocarcinoma of lung"[All Fields] OR ("lung"[All Fields] AND "adenocarcinoma"[All Fields]) OR "lung adenocarcinoma"[All Fields]) OR (("lung"[MeSH Terms] OR "lung"[All Fields]) AND ("carcinoma, squamous cell"[MeSH Terms] OR ("carcinoma"[All Fields] AND "squamous"[All Fields] AND "cell"[All Fields]) OR "squamous cell carcinoma"[All Fields] OR ("squamous"[All Fields] AND "cell"[All Fields] AND "carcinoma"[All Fields])))) AND ("afatinib"[MeSH Terms] OR "afatinib"[All Fields] OR ("afatinib"[MeSH Terms] OR "afatinib"[All Fields] OR "gilotrif"[All Fields]) OR ("afatinib"[MeSH Terms] OR "afatinib"[All Fields] OR "bibw2992"[All Fields])) |
| *Embase（n=3846）* | ('non small cell lung cancer'/exp OR 'non small cell lung cancer' OR 'non small cell lung carcinoma'/exp OR 'non small cell lung carcinoma' OR 'nsclc' OR 'lung adenocarcinoma'/exp OR 'lung adenocarcinoma') AND ('afatinib' OR 'gilotrif' OR 'bibw2992') |
| *Cochrane Library（n=28）* | #1 MeSH descriptor: [Afatinib] explode all trees  #2 MeSH descriptor: [Carcinoma, Non-Small-Cell Lung] explode all trees  #3 #1 AND #2 |
| *ClinicalTrials.gov（n=92）* | Afatinib \| Non Small Cell Lung Cancer |
| *CNKI（n=96）* | （主题：阿法替尼）AND（主题：非小细胞肺癌） |
| *WanFang（n=129）* | 主题:(阿法替尼) and 主题:(非小细胞肺癌) |

Supplementary Table B Risk-of-bias assessment for included cohort studies using the modified Newcastle–Ottawa scale

| Study | From the same population | Assessment of exposure | Outcome not present at start | Adjustment | Assessment of prognostic factors | Assessment of outcome | Adequate follow-up | Similar co-interventions |
| --- | --- | --- | --- | --- | --- | --- | --- | --- |
| Arrieta, 2015^15^ | Definitely Yes | Definitely Yes | Definitely Yes | Probably Yes | Definitely Yes | Definitely Yes | Probably Yes | Definitely Yes |
| Halmos, 2019^26^ | Definitely Yes | Definitely Yes | Probably Yes | Definitely Yes | Definitely Yes | Definitely Yes | Definitely Yes | Definitely Yes |
| Ko, 2020^16^ | Definitely Yes | Definitely Yes | Definitely Yes | Probably Yes | Probably No | Definitely Yes | Definitely Yes | Probably Yes |
| Lim, 2018^17^ | Definitely Yes | Definitely Yes | Definitely Yes | Probably No | Probably Yes | Definitely Yes | Definitely Yes | Definitely Yes |
| Moran, 2017^18^ | Definitely Yes | Definitely Yes | Definitely Yes | Probably Yes | Probably No | Definitely Yes | Probably Yes | Definitely Yes |
| Ninomiya, 2018^19^ | Definitely Yes | Definitely Yes | Definitely Yes | Probably Yes | Probably Yes | Definitely Yes | Definitely Yes | Definitely Yes |
| Tamura, 2019^20^ | Definitely Yes | Definitely Yes | Definitely Yes | Probably Yes | Definitely Yes | Definitely Yes | Definitely Yes | Definitely Yes |
| Tan, 2018^21^ | Definitely Yes | Definitely Yes | Definitely Yes | Probably Yes | Definitely Yes | Definitely Yes | Definitely Yes | Definitely Yes |
| Tanaka, 2018^22^ | Definitely Yes | Definitely Yes | Definitely Yes | Probably Yes | Probably No | Definitely Yes | Definitely Yes | Definitely Yes |
| Wang, 2019^23^ | Definitely Yes | Definitely Yes | Definitely Yes | Definitely Yes | Definitely Yes | Definitely Yes | Definitely Yes | Probably Yes |
| Wei, 2019^24^ | Definitely Yes | Definitely Yes | Definitely Yes | Probably Yes | Probably Yes | Definitely Yes | Definitely Yes | Probably Yes |
| Yang, 2017^25^ | Definitely Yes | Definitely Yes | Definitely Yes | Probably No | Definitely Yes | Definitely Yes | Definitely Yes | Probably Yes |

**Appendix B:** Supplementary materials Analysis results of PFS in patients with 30 mg and 40 mg afatinib

Figure S1 Analysis results of PFS in patients with non-small cell lung cancer who used 30 mg and 40 mg afatinib

Figure S2: Analysis results of PFS in patients with adenocarcinoma who used 30 mg and 40 mg afatinib

Figure S3 Analysis results of PFS in patients with non-small cell lung cancer who first-line used 30 mg and 40 mg afatinib

Figure S4: Analysis results of PFS in patients with adenocarcinoma who first-line used 30 mg and 40 mg afatinib

Figure S5 Analysis results of PFS in patients with stage Ⅳ non-small cell lung cancer who first-line used 30 mg and 40 mg afatinib

Figure S6: Analysis results of PFS in patients with stage Ⅳ adenocarcinoma who first-line used 30 mg and 40 mg afatinib
